# Vesiclepedia: A Compendium for Extracellular Vesicles with Continuous Community Annotation

**DOI:** 10.1371/journal.pbio.1001450

**Published:** 2012-12-18

**Authors:** Hina Kalra, Richard J. Simpson, Hong Ji, Elena Aikawa, Peter Altevogt, Philip Askenase, Vincent C. Bond, Francesc E. Borràs, Xandra Breakefield, Vivian Budnik, Edit Buzas, Giovanni Camussi, Aled Clayton, Emanuele Cocucci, Juan M. Falcon-Perez, Susanne Gabrielsson, Yong Song Gho, Dwijendra Gupta, H. C. Harsha, An Hendrix, Andrew F. Hill, Jameel M. Inal, Guido Jenster, Eva-Maria Krämer-Albers, Sai Kiang Lim, Alicia Llorente, Jan Lötvall, Antonio Marcilla, Lucia Mincheva-Nilsson, Irina Nazarenko, Rienk Nieuwland, Esther N. M. Nolte-'t Hoen, Akhilesh Pandey, Tushar Patel, Melissa G. Piper, Stefano Pluchino, T. S. Keshava Prasad, Lawrence Rajendran, Graca Raposo, Michel Record, Gavin E. Reid, Francisco Sánchez-Madrid, Raymond M. Schiffelers, Pia Siljander, Allan Stensballe, Willem Stoorvogel, Douglas Taylor, Clotilde Thery, Hadi Valadi, Bas W. M. van Balkom, Jesús Vázquez, Michel Vidal, Marca H. M. Wauben, María Yáñez-Mó, Margot Zoeller, Suresh Mathivanan

**Affiliations:** 1 Department of Biochemistry, La Trobe Institute for Molecular Science, La Trobe University, Melbourne, Victoria, Australia; 2 Cardiovascular Medicine, Brigham and Women's Hospital, Harvard Medical School, Boston, Massachusetts, United States of America; 3 Tumor Immunology Programme, German Cancer Research Center, Heidelberg, Germany; 4 Department of Medicine, Yale Medical School, New Haven, Connecticut, United States of America; 5 Department of Microbiology, Biochemistry, and Immunology, Morehouse School of Medicine, Atlanta, Georgia, United States of America; 6 IVECAT, LIRAD-BST, Institut d'Investigació Germans Trias i Pujol, Dept de Biologia Cellular, Fisiologia i Immunologia, Universitat Autònoma de Barcelona, Badalona, Spain; 7 Department of Neurology, Massachusetts General Hospital, and Neuroscience Program, Harvard Medical School, Boston, Massachusetts, United States of America; 8 Department of Neurobiology, University of Massachusetts Medical School, Worcester, Massachusetts, United States of America; 9 Department of Genetics, Cell- and Immunobiology, Semmelweis University, Budapest, Hungary; 10 Department of Internal Medicine, Centre for Molecular Biotechnology and Centre for Research in Experimental Medicine, Torino, Italy; 11 Institute of Cancer & Genetics, School of Medicine, Cardiff University, Velindre Cancer Centre, Whitchurch, Cardiff, United Kingdom; 12 Department of Cell Biology, Harvard Medical School, Boston, Massachusetts, United States of America; 13 Immune Disease Institute and Program in Cellular and Molecular Medicine at Boston Children's Hospital, Boston, Massachusetts, United States of America; 14 Metabolomics Unit, CIC bioGUNE, CIBERehd, Technology Park of Bizkaia, Derio, Bizkaia, Spain; 15 IKERBASQUE, Basque Foundation for Science, Bilbao, Spain; 16 Translational Immunology Unit, Department of Medicine Solna, Karolinska Institutet, Stockholm, Sweden; 17 Department of Life Science, Pohang University of Science and Technology, Pohang, Republic of Korea; 18 Center of Bioinformatics, Institute of Interdisciplinary Studies, University of Allahabad, Allahabad, India; 19 Institute of Bioinformatics, Bangalore, India; 20 Laboratory of Experimental Cancer Research, Department of Radiation Oncology and Experimental Cancer Research, Ghent University Hospital, Ghent, Belgium; 21 Department of Biochemistry and Molecular Biology, Bio21 Molecular Science and Biotechnology Institute, The University of Melbourne, Parkville, Australia; 22 Cellular and Molecular Immunology Research Centre, Faculty of Life Sciences, London Metropolitan University, London, United Kingdom; 23 Department of Urology, Erasmus Medical Centre, Rotterdam, The Netherlands; 24 Department of Molecular Cell Biology, Johannes Gutenberg University Mainz, Mainz, Germany; 25 A*STAR Institute of Medical Biology and Department of Surgery, Yong Loo Lin School of Medicine, National University of Singapore, Singapore; 26 Department of Biochemistry, Institute for Cancer Research, Oslo University Hospital-The Norwegian Radium Hospital, Oslo, Norway; 27 Krefting Research Centre, Department of Internal Medicine and Clinical Nutrition, Institute of Medicine, Sahlgrenska Academy, University of Gothenburg, Gothenburg, Sweden; 28 Área de Parasitología, Departamento de Biología Celular y Parasitología, Universitat de València, Burjassot (Valencia), Spain; 29 Department of Clinical Microbiology/Clinical Immunology, Umeå University, Umeå, Sweden; 30 Department of Environmental Health Sciences, University Medical Center Freiburg, Freiburg, Germany; 31 Department of Clinical Chemistry, Academic Medical Center, Amsterdam, The Netherlands; 32 Department of Biochemistry & Cell Biology, Faculty of Veterinary Medicine, Utrecht University, Utrecht, The Netherlands; 33 McKusick-Nathans Institute of Genetic Medicine, Johns Hopkins University School of Medicine, Baltimore, Maryland, United States of America; 34 Department of Biological Chemistry, Johns Hopkins University School of Medicine, Baltimore, Maryland, United States of America; 35 Department of Oncology and Pathology, Johns Hopkins University School of Medicine, Baltimore, Maryland, United States of America; 36 Mayo Clinic, Jacksonville, Florida, United States of America; 37 Department of Internal Medicine, Division of Pulmonary, Allergy, Critical Care and Sleep Medicine, Davis Heart & Lung Research Institute, The Ohio State University, Columbus, Ohio, United States of America; 38 Center for Brain Repair and Wellcome Trust-MRC Stem Cell Institute, Department of Clinical Neurosciences, University of Cambridge, Cambridge, United Kingdom; 39 Systems and Cell Biology of Neurodegeneration, Division of Psychiatry Research, University of Zurich, Zurich, Switzerland; 40 Institut Curie, Paris, France; 41 Cancer Researaud, Toulouse, France; 42 Department of Chemistry, Department of Biochemistry and Molecular Biology, Michigan State University, East Lansing, Michigan, United States of America; 43 Servicio de Inmunologia, Hospital de la Princesa, Universidad Autonoma Madrid, Madrid, Spain; 44 Laboratory of Clinical Chemistry and Haematology, University Medical Center Utrecht, Utrecht, The Netherlands; 45 Department of Biosciences, Division of Biochemistry and Biotechnology, University of Helsinki, Finland; 46 Institute for Biotechnology, University of Aalborg, Denmark; 47 Department of Biochemistry and Cell Biology, Faculty of Veterinary Medicine and Institute of Biomembranes, Utrecht University, Utrecht, The Netherlands; 48 Department of Obstetrics, Gynecology and Women's Health and James Graham Brown Cancer Center, University of Louisville School of Medicine, Louisville, Kentucky, United States of America; 49 Institut Curie Centre de Recherche, Paris, France; 50 INSERM U932, Paris, France; 51 Department of Rheumatology and Inflammation Research, Sahlgrenska Academy, University of Gothenburg, Gothenburg, Sweden; 52 Department of Nephrology and Hypertension, University Medical Center Utrecht, Utrecht, The Netherlands; 53 Cardiovascular Proteomics Laboratory, Centro Nacional de Investigaciones Cardiovasculares (CNIC), Madrid, Spain; 54 UMR 5235 CNRS-University Montpellier II, Montpellier, France; 55 Department of Biochemistry & Cell Biology, Faculty of Veterinary Medicine, Life Sciences, Utrecht University, Utrecht, The Netherlands; 56 Unidad de Investigación, Hospital Santa Cristina, Instituto de Investigación Sanitaria Princesa, Madrid, Spain; 57 Department of Tumor Cell Biology, University Hospital of Surgery, Heidelberg, Germany

## Abstract

Extracellular vesicles (EVs) are membraneous vesicles released by a variety of cells into their microenvironment. Recent studies have elucidated the role of EVs in intercellular communication, pathogenesis, drug, vaccine and gene-vector delivery, and as possible reservoirs of biomarkers. These findings have generated immense interest, along with an exponential increase in molecular data pertaining to EVs. Here, we describe Vesiclepedia, a manually curated compendium of molecular data (lipid, RNA, and protein) identified in different classes of EVs from more than 300 independent studies published over the past several years. Even though databases are indispensable resources for the scientific community, recent studies have shown that more than 50% of the databases are not regularly updated. In addition, more than 20% of the database links are inactive. To prevent such database and link decay, we have initiated a continuous community annotation project with the active involvement of EV researchers. The EV research community can set a gold standard in data sharing with Vesiclepedia, which could evolve as a primary resource for the field.

## Abstract 2

Vesiclepedia is a community-annotated compendium of molecular data on extracellular vesicles.

## Introduction

A growing body of research has implicated extracellular vesicles (EVs), membraneous sacs released by a variety of cells, in diverse physiological and patho-physiological conditions [1]–[9]. They can be detected in body fluids including blood plasma, urine, saliva, amniotic fluid, breast milk, and pleural ascites [10]–[13], and contain proteins, lipids, and RNA representative of the host cell [14]–[18]. Though a definitive categorization is yet to be achieved [19], EVs can be broadly classified into three main classes, based on the mode of biogenesis: (i) ectosomes (also referred to as shedding microvesicles), (ii) exosomes, and (iii) apoptotic bodies (ABs) (see Box 1).

**Box 1. Categories of EVs Based on the Mode of Biogenesis**

**Ectosomes or shedding microvesicles**: Ectosomes are large EVs ranging between 50–1,000 nm in diameter [1]. They are shed from cells by outward protrusion (or budding) of a plasma membrane (PM) followed by fission of their membrane stalk [3],[5]. Ectosomes are released by a variety of cells including tumour cells, polymorphonuclear leucocytes, and aging erythrocytes [5]. The expression of phosphatidylserine (PS) on the membrane surface has been shown to be one of the characteristic features of ectosomes [1],[5].

**Exosomes**: Exosomes are small membranous vesicles of endocytic origin ranging from 40–100 nm in diameter [1],[42]. The density of exosomes varies from 1.10–1.21 g/ml and the commonly found markers of exosomes are Alix, TSG101, tetraspanins, and heat shock proteins [10]. The biogenesis of exosomes begins with the internalisation of molecules via endocytosis [42]. Once internalised, endocytosed molecules are either recycled to the PM or trafficked to multivesicular bodies (MVBs) [3]. The “exocytic” fate of MVBs results in their exocytic fusion with the PM, resulting in the release of intraluminal vesicles into the extracellular microenvironment as exosomes [43].

**Apoptotic bodies**: ABs are released from fragmented apoptotic cells and are 50–5,000 nm in diameter [1]. ABs are formed about during the process of programmed cell death or apoptosis, and represent the fragments of dying cells [3]. Similar to ectosomes, the expression of PS on the membrane surface has been shown to be a key characteristic of ABs [1],[5].

Recent studies have highlighted the role of EVs in intercellular communication [20]–[22], vaccine and drug delivery [23]–[25], and suggested a potential role in gene vector therapy [26] and as disease biomarkers [27]. More than three decades of research has advanced our basic understanding of these extracellular organelles and has generated large amounts of multidimensional data [14],[17]. Whilst most of the data are presented in the context of the biological findings/technical development and are mentioned in the inline text of the published article, a vast majority are often placed as supplementary information or not provided [28],[29]. Importantly, none of the molecular data in published articles is easily searchable [28]. With the immense interest in EVs and advances in high-throughput techniques, the data explosion will only increase. An online compendium of heterogeneous data will help the biomedical community to exploit the publicly available datasets and accelerate biological discovery [30].

## ExoCarta and Need for an EV Database

Existing databases are not comprehensive. For example, ExoCarta (http://www.exocarta.org), a database for molecular data (proteins, RNA, and lipids) identified in exosomes, catalogs only exosomal studies (as reported by the authors) [31]. Described initially in 2009 [32], the database has been visited by more than 16,000 unique users [33]. However, only exosomal studies (as reported by the authors) are catalogued in ExoCarta. With the confusion in terminologies and inefficiency of the purification protocols to clearly segregate each class of EVs [1],[19], it is critical to build a repository with data from all classes of EVs to understand more about the molecular repertoire of the various classes of EVs and their biological functions. This was the rationale for starting the Vesiclepedia online compendium for EVs.

## Vesiclepedia

Vesiclepedia (http://www.microvesicles.org) is a manually curated compendium that contains molecular data identified in all classes of EVs, including AB, exosomes, large dense core vesicles, microparticles, and shedding microvesicles. The main criterion for manual curation was the presence of these vesicles in the extracellular microenvironment (EVs) as approved by the investigators who undertook the research. At this juncture, the EVs are named as per the curated article or submitting author, as the nomenclature is yet to be standardized [19]. Vesiclepedia was built using ZOPE, an open source content management system. Python a portable, interpreted, object oriented programming language was used in the three-tier system to connect the web interface with a MySQL database. Users can query or browse through proteins, lipids, and RNA molecules identified in EVs. Selecting a gene of interest directs the user to a gene/molecule page with information on the gene, its external references to other primary databases, experiment description of the study that identified the molecule, gene ontology based annotations, protein-protein interactions, and a graphical display of such network with relevance to molecules identified in EVs. Gene ontology annotations of molecular functions, biological process, and subcellular localization were retrieved from Entrez Gene [34] and mapped onto the proteins/mRNA identified in EVs. Under the experiment description, the sample source including the tissue name or cell line name, EV isolation procedures, and floatation gradient density as reported in the study are provided to the users. EV proteins are mapped onto their protein physical interactors along with the protein interaction identification method and PubMed identifier. Protein-protein interaction data was obtained from HPRD [35],[36], BioGRID [37], and Human Proteinpedia [38].

## Database Issues and Community Annotation

Though biological databases are indispensable resources for effective scientific research, it has to be noted that more than 20% of the database links are non-existent after their initial publication [39]–[41]. More than 50% of the databases are never updated reducing their usability [39], primarily due to the lack of continuous funding to maintain and update these resources. At this juncture, funding for databases is largely non-existent in many parts of the world. To overcome funding-related limitations and to keep the database updated, it is essential to involve the scientific community in annotating the data. Community annotation will significantly ease the burden of the curators who maintain and update the databases. Whilst community annotation is the permanent solution to keep the database updated, it seldom happens without a clear and transparent mechanism. In addition, the system has to ensure continuous deposition of data and “not just once” uploads. It has to be noted that data annotation can be regulated at two levels: (i) principal investigators voluntarily contributing data and (ii) peer-reviewed journals mandating data deposition before publication. Currently available community annotation tools don't have a continuous data deposition arrangement with an investigator. Additionally, only few journals mandate the deposition of data to public repositories before acceptance of a manuscript. To this end, we have initiated a community annotation project through Vesiclepedia that involves members of the EV research community (53 laboratories from 20 countries: Table 1).

**Table 1:** Vesiclepedia statistics.

Statistics	*n*
EV studies	341
Protein entries	35,264
mRNA entries	18,718
miRNA entries	1,772
Lipid molecules	342
Participating laboratories	53
Countries	20

Community annotation via Vesiclepedia happens through the founding members who agree to the conditions listed in Box 2. All of the members are listed in the credits page (http://www.microvesicles.org/credits).

**Box 2. Conditions to Become a Member of Vesiclepedia Community Annotation**

1, Agree to submit datasets pertaining to EVs to Vesiclepedia before or after publication on a continuous basis

2, When reviewing articles, mandate/request investigators to submit datasets on EVs to Vesiclepedia

**Figure 1:**
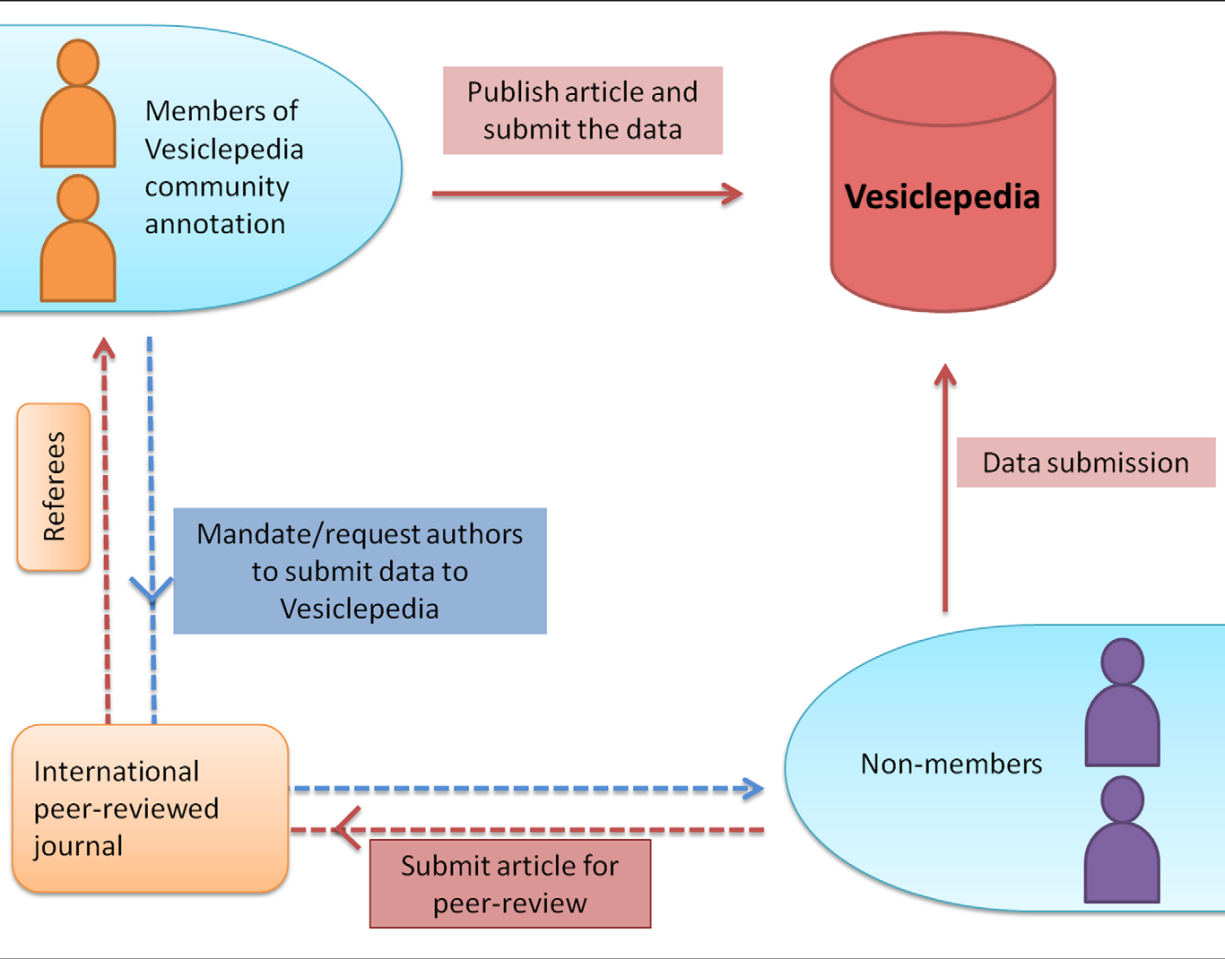
A schematic of Vesiclepedia community annotation. Based on the agreement of community participation, members will submit their data automatically to Vesiclepedia before and after publication. Non-members submitting their research findings for peer-review through international journals might find some of the Vesiclepedia members as referees who will request/mandate the authors to submit the data to Vesiclepedia. Alternatively, a non-member can also be appointed as a referee in which case the data might not be submitted to Vesiclepedia. A non-member can also submit data directly to Vesiclepedia.

On the basis of the agreement of community participation, members will submit their data automatically to Vesiclepedia before or after publication (Figure 1). Non-members submitting their research findings for peer-review through international journals might find the Vesiclepedia members as referees who will request/mandate the authors to submit the data to Vesiclepedia. By instituting this mechanism the datasets will be continuously deposited to Vesiclepedia. However, a non-member can also be appointed as a referee in which case the data might not be submitted to Vesiclepedia. The Vesiclepedia-data capture team will work along with the researchers to make the data submission as easy as possible. Detailed information on the format of data required for submission is provided in the Vesiclepedia webpage (http://www.microvesicles.org/data_submission). Currently, Vesiclepedia comprises 35,264 protein, 18,718 mRNA, 1,772 miRNA, and 342 lipid entries (Table 1). All of these data were obtained from 341 independent studies that were published over the past several years.

## Conclusions and Future Directions

ExoCarta will be active even after the release of Vesiclepedia and will become a primary resource for high-quality exosomal datasets. Data deposited to ExoCarta can also be accessed through Vesiclepedia; however, only high quality exosomal datasets deposited to Vesiclepedia can be accessed through ExoCarta. With the launch of Vesiclepedia, we expect to have an organised data deposition mechanism. We expect active participation from the EV research community, along with the addition of new members and numerous heterogeneous datasets. All datasets submitted by EV researchers will be listed in the credits page along with the investigator details.

## Abbreviations

AB: apoptotic body
EV: extracellular vesicle
